# Association between blood urea nitrogen to serum albumin ratio and in-hospital mortality in critical patients with diabetic ketoacidosis: a retrospective analysis of the eICU database

**DOI:** 10.3389/fendo.2024.1411891

**Published:** 2024-06-27

**Authors:** Hua Chen, Yufei Wang, Rong Ji, Minghui Li

**Affiliations:** ^1^ Center of Cardiovascular Medicine, Inner Mongolia People’s Hospital, Hohhot, China; ^2^ Graduate School, Inner Mongolia Medical University, Hohhot, China; ^3^ State Key Laboratory of Cardiovascular Disease, Department of Cardiology, Fuwai Hospital, National Center for Cardiovascular Disease, Chinese Academy of Medical Science and Peking Union Medical College, Beijing, China

**Keywords:** blood urea nitrogen to serum albumin ratio, in-hospital mortality, intensive care unit, diabetic ketoacidosis, eICU database

## Abstract

**Background:**

This study aimed to investigate the association between blood urea nitrogen to serum albumin ratio (BAR) and the risk of in-hospital mortality in patients with diabetic ketoacidosis.

**Methods:**

A total of 3,962 diabetic ketoacidosis patients from the eICU Collaborative Research Database were included in this analysis. The primary outcome was in-hospital death.

**Results:**

Over a median length of hospital stay of 3.1 days, 86 in-hospital deaths were identified. One unit increase in LnBAR was positively associated with the risk of in-hospital death (hazard ratio [HR], 1.82 [95% CI, 1.42–2.34]). Furthermore, a nonlinear, consistently increasing correlation between elevated BAR and in-hospital mortality was observed (P for trend =0.005 after multiple-adjusted). When BAR was categorized into quartiles, the higher risk of in-hospital death (multiple-adjusted HR, 1.99 [95% CI, (1.1–3.6)]) was found in participants in quartiles 3 to 4 (BAR≥6.28) compared with those in quartiles 1 to 2 (BAR<6.28). In the subgroup analysis, the LnBAR-hospital death association was significantly stronger in participants without kidney insufficiency (yes versus no, P-interaction=0.023).

**Conclusion:**

There was a significant and positive association between BAR and the risk of in-hospital death in patients with diabetic ketoacidosis. Notably, the strength of this association was intensified among those without kidney insufficiency.

## 1 Introduction

Diabetic ketoacidosis (DKA) is one of the most serious acute metabolic complications of diabetes (1, 2). DKA was characterized by hyperglycemia, ketosis, and metabolic acidosis. The diagnosis of DKA is typically based on the presence of hyperglycemia (blood glucose >250 mg/dL), ketonemia or ketonuria, and a high anion gap metabolic acidosis (serum bicarbonate <18 mEq/L and/or arterial pH <7.3) (3, 4).

Among individuals with diabetes, 10% of deaths are caused by confirmed or probable DKA or coma (4). Patients diagnosed with DKA or those experiencing recurrent DKA are at a significantly higher risk of all-cause mortality during their hospital stay (3). Moreover, these patients have a higher long-term mortality rate after discharge (5, 6). Therefore, it is crucial to identify precise and straightforward prognostic indicators.

Blood urea nitrogen to serum albumin ratio (BAR), as an innovative risk factor, is readily identifiable and easily accessible. It has been established as a prognostic marker for severe chronic obstructive pulmonary disease, sepsis, and cerebral hemorrhage (7–9). Due to hyperglycemia, DKA patients frequently exhibit osmotic diuresis, volume depletion, electrolyte imbalances, and a catabolic state. These conditions frequently result in complications such as dehydration, impaired renal function, and malnutrition (10, 11). The BUN level and serum albumin level are closely related to these conditions and complications (12–14). Consequently, BAR, a novel indicator that combines both indicators, may be a valuable and easily accessible prognostic indicator for DKA. However, relevant studies are lacking.

The aim of this study was to investigate the predictive value of BAR in relation to in-hospital mortality among patients with DKA. The data used in this study were obtained from the eICU database.

## 2 Materials and methods

Research data were extracted from the eICU Collaborative Research Database, which included medical information on 200,859 ICU admissions for 139,367 unique patients who were admitted to 335 ICUs across 208 hospitals in the USA between 2014 and 2015 (15). The author Li Minghui has completed PhysioNet training and was authorized to access the eICU database (Record ID: 55159033) (16).

### 2.1 Study population

**Figure 1:**
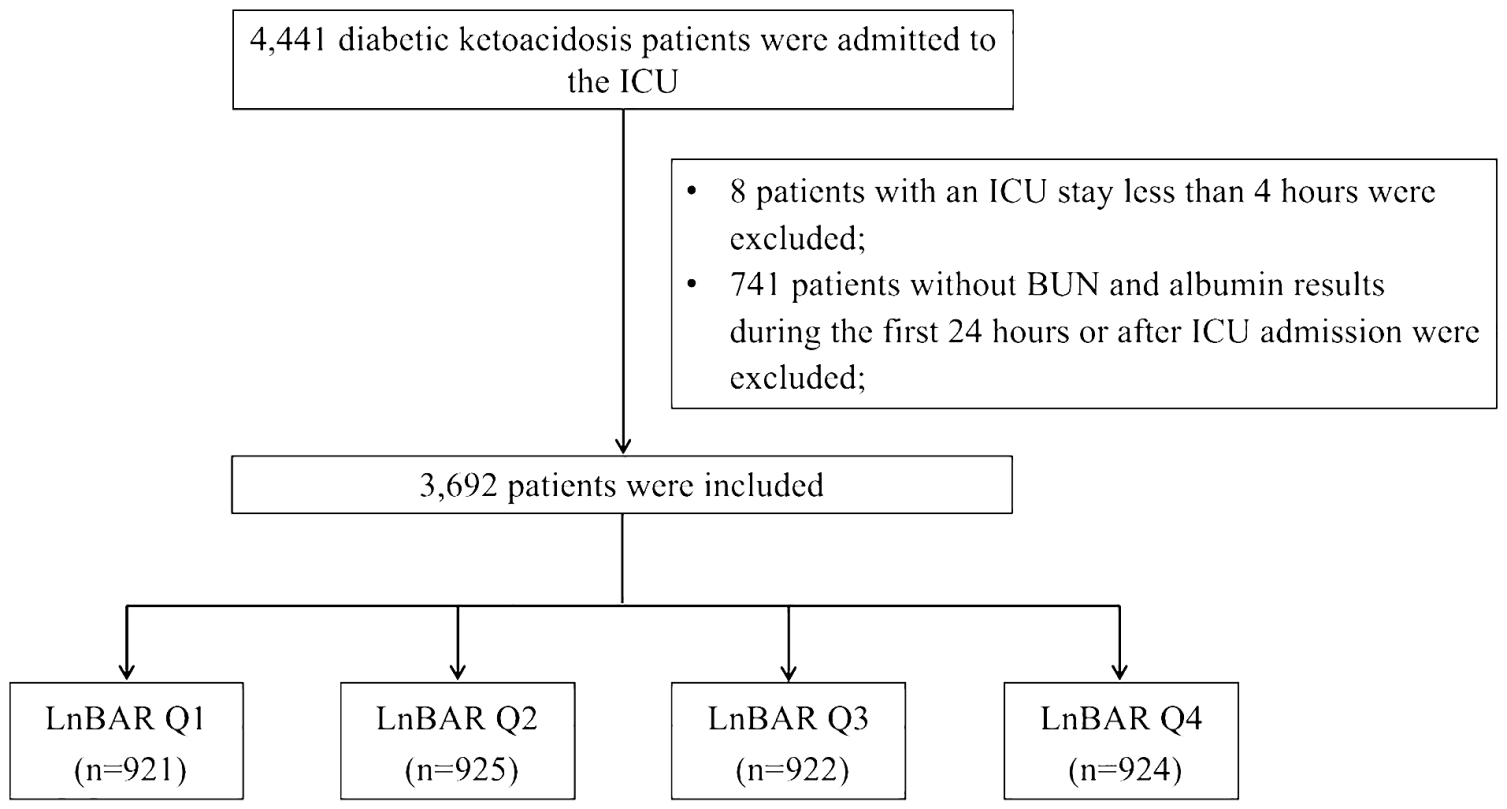
Flow chart of study population selection. ICU, intensive care unit; BUN, blood urea nitrogen; LnBAR, the natural logarithm of blood urea nitrogen to serum albumin ratio.

A total of 4,441 DKA patients were initially identified by looking for International Classification of Diseases version 9 (ICD-9) code 250.1 (Diabetes with ketoacidosis) and the text “DKA/diabetic ketoacidosis” in the diagnosis datafile. The exclusion criteria included: (1) discharged within 4 hours after ICU admission; (2) no laboratory record of BUN or albumin results during the first 24 hours or after ICU admission. For patients with multiple ICU admissions, only the first admission record was kept. A total of 3,962 DKA patients entered the study cohort (**Figure 1**).

### 2.2 Selection of lab test results

In one ICU stay, a lab item could be repeatedly tested as treatment proceeded. To capture the value of lab items most representative of a patient’s baseline status at the time of ICU admission, the test result of a lab item which record time was closest to the ICU admission time and within 24 h of ICU admission was used. Lab data have been technically validated and checked for integrity following a series of procedures stated in the database document (17). To maintain statistical power and diminish bias that might occur after subjects with missing data were excluded from analyses, we used multivariate multiple imputation with chained equations to impute missing values (18).

### 2.3 Exposure and outcome measurements

Due to the positively skewed distribution of BAR with a long right tail, the natural logarithm of BAR (LnBAR) of patients measured at the time closest to the ICU admission time and within 24 h of ICU admission was employed as the exposure of this study. The “labResultOffset” column of the “lab” table in the eICU database stored the interval between a patient’s ICU admission time and the time a biomarker’s lab value was drawn. By joining the “lab” table with patient information and selecting the minimum offset value, the exact blood urea nitrogen and serum albumin value measured closest to the ICU admission of each patient was identified.

The study outcome was in-hospital death. In-hospital death was defined as death occurring on or before the day of hospital discharge. It was identified by examining the “hospitalDischargeStatus” and “unitDischargeStatus” of the patient table. The general introduction of table structure and database schema can be freely accessed at https://eicu-crd.mit.edu.

### 2.4 Identification of covariates

The inclusion of covariables is based on the following principles: (1) Based on a series of existing publications (19–24); (2) Covariables change the impact estimates of the correlation between DKA hospitalization mortality by 10% or more; or (3) Clinical experience. Covariates with multiple collinearities were excluded from the analysis. Body mass index (BMI) was calculated as admission weight in kilograms divided by the square value of admission height in meters. Ethnicity was categorized as White, Black, Asian and others, as documented in the database. Comorbidities diagnosed during the ICU stay were identified by examining ICD-9 codes and diagnosis text of the Philips eCareManager system from the database. The comorbidities included in this research were diabetes type, hypertension, acute myocardial infarction, heart failure, infectious disease, chronic kidney disease, neurological diseases, dialysis requirement, and history of invasive ventilation use (IVU). The vital signs including heart rate and mean arterial pressure were included. Serum electrolyte levels including blood sodium, blood potassium, blood calcium, and blood chloride were included. Indicators of liver and kidney function including eGFR, alanine transferase (ALT), and aspartate aminotransferase (AST) were included. The level of blood glucose was included. Blood gas parameters including bicarbonate, anion gap, PaO_2_, and PaCO_2_ were included. White blood cell counts and hemoglobin levels in the blood were included. Sepsis was included. The treatment histories of dialysis and invasive ventilation were included. Furthermore, Glasgow Coma Scale (GCS) was included to identify patients at high risk.

### 2.5 Statistical analysis

Baseline characteristics of the study cohort were stratified by the quartile of their baseline LnBAR levels. Continuous data were presented as the mean (standard deviation [SD]) for normally distributed data and the median (interquartile range [IQR]) for skewed data. Categorical data were presented as the total number of an item with column percentage (n [%]) unless otherwise specified. Crude comparisons between strata were performed using Kruskal-Wallis rank test for continuous data and chi-square tests for categorical variables.

Univariate and multivariate Cox regression models were used to assess the hazard ratio (HR) of LnBAR with in-hospital mortality. In the crude model, no variables were adjusted for during the analysis. In Model 1, the potential confounded factors including age, gender, and ethnicity were incorporated into the adjustment process. Model 2 expanded on Model 1, accounting for a more comprehensive list of parameters. This included BMI, heart rate, and MAP. The adjusted logarithmic relative risks (logRRs) of in-hospital death plotted against LnBAR were visualized, with a fitted smoothed curve to represent the trend. Non-linearity of these associations was tested with a likelihood ratio test. we used a recursive algorithm to calculate the inflection points. We also graphically represented the adjusted log RRs for in-hospital death across the continuous range of LnBAR using three separate plots stratified by sex, age categories (<65 years vs. ≥65 years), and kidney insufficiency status (yes vs. no).

Then, the time-dependent receiver operating characteristic (TDROC) curves were used to examine the association of the BAR with in-hospital mortality. The areas under the TDROC curves were used to compare the predictive value of BAR and the predictive value of BUN.

Sensitivity analysis was performed. (1) To authenticate the reliability of the data following multiple imputations, the percentage accounted for by the missing variables were computed. Subsequently, we orchestrated a comparison of the distributions of the missing variables prior to and following the implementation of multiple imputation. (2) Moreover, multivariate Cox regression was performed on complete case data as well as the dataset post multiple imputation separately. (3) To investigate a potential role of serum electrolyte levels, liver and kidney indices, diabetes type and blood glucose level, blood gas parameters, WBC count and hemoglobin level, sepsis and infectious diseases, comorbidities, and GCS with any of the observed associations, we further adjusted for serum electrolyte levels (including blood sodium, blood potassium, blood calcium, and blood chloride), indicators of liver and kidney function (eGFR, ALT, and AST), diabetes type and blood glucose level, blood gas parameters (blood bicarbonate, anion gap, PaO2 and chloride), indicators of liver and kidney function (eGFR, ALT, and AST), diabetes type and blood glucose level, blood gas parameters (blood bicarbonate, anion gap, PaO2, PaCO2), WBC count and hemoglobin level, sepsis and infectious disease, comorbidities (neurological disease, acute myocardial infarction, hypertension, heart failure, chronic renal failure), and GCS. (4) The main analyses were repeated on data set without participants with eGFR < 30 ml/min/1.73m^2^. (5) The areas under the TDROC curves were employed to assess the predictive value of BAR in comparison to that of other direct markers of acidosis, including serum anion gap, serum bicarbonate, and blood glucose. (6) As an observational study, we included a STROBE (STrengthening the Reporting of OBservational studies in Epidemiology) checklist of items in the sensitivity analyses to ensure the reproducibility of the reporting. The analyses were done using the statistical packages R v.4.2.0 (R Foundation for Statistical Computing; http://www.r-project.org). A two-sided P value < 0.05 was considered statistically significant.

## 3 Results

### 3.1 Baseline characteristics

Over a median length of hospital stay of 3.1 days, 86 in-hospital deaths were identified. Significant differences in baseline characteristics including age, sex, ethnicity, BMI, heart rate, MAP, medical history, laboratory results, IVU, and dialysis were found among baseline BAR quartiles (**Table 1**).

**Table 1:** Baseline characteristics of participants based on BAR quartiles.

Variables	All patients	BAR quartiles				*P*-value
		Q1 (<4.07)	Q2 (4.07-6.28)	Q3 (6.28-11.58)	Q4 (≥11.58)	
	N=3692	N=921	N=925	N=922	N=924	
**Age, years**	43.6 ± 17.3	33.6 ± 12.7	37.5 ± 15.1	48.9 ± 16.4	54.4 ± 15.9	<0.001
**Male, n (%)**	1793 (48.6)	398 (43.2)	429 (46.4)	478 (51.8)	488 (52.8)	<0.001
**Ethnicity, n (%)**						0.002
African American	582 (15.8)	162 (17.6)	130 (14.1)	135 (14.6)	155 (16.8)	
Caucasian	2578 (69.8)	598 (64.9)	684 (73.9)	660 (71.6)	636 (68.8)	
Asian and others	532 (14.4)	161 (17.5)	111 (12.0)	127 (13.8)	133 (14.4)	
**BMI, kg/m^2^**	26.1 ± 7.4	25.9 ± 7.5	25.4 ± 6.8	26.5 ± 7.6	26.6 ± 7.5	<0.001
**Heart rate, bpm**	114.2 ± 23.9	115.1 ± 22.4	116.3 ± 22.4	113.9 ± 23.0	111.5 ± 27.0	<0.001
**MAP, mmHg**	82.0 ± 36.2	79.4 ± 30.6	77.2 ± 31.2	84.1 ± 38.2	87.3 ± 42.8	<0.001
Comorbidities during ICU stay, n (%)						
Infectious disease	244 (6.6)	26 (2.8)	37 (4.0)	56 (6.1)	125 (13.5)	<0.001
Neurological diseases	227 (6.1)	31 (3.4)	26 (2.8)	68 (7.4)	102 (11.0)	<0.001
AMI	95 (2.6)	4 (0.4)	5 (0.5)	31 (3.4)	55 (6.0)	<0.001
Hypertension	409 (11.1)	61 (6.6)	75 (8.1)	106 (11.5)	167 (18.1)	<0.001
CHF	85 (2.3)	0 (0.0)	5 (0.5)	25 (2.7)	55 (6.0)	<0.001
CKD	294 (8.0)	10 (1.1)	26 (2.8)	69 (7.5)	189 (20.5)	<0.001
Diabetes						<0.001
Type I	139 (3.8)	35 (3.8)	49 (5.3)	35 (3.8)	20 (2.2)	
Type II	69 (1.9)	13 (1.4)	8 (0.9)	20 (2.2)	28 (3.0)	
Unknown	3484 (94.4)	873 (94.8)	868 (93.8)	867 (94.0)	876 (94.8)	
Laboratory results						
BUN, mg/dl	24.0 (16.0-38.0)	12.0 (9.0-15.0)	20.0 (17.0-23.0)	30.0 (25.0-35.0)	56.0 (44.0-74.0)	<0.001
Sodium, mEq/l	132.0 ± 6.9	133.2 ± 4.8	132.3 ± 5.4	132.2 ± 6.5	130.3 ± 9.6	<0.001
Potassium, mEq/l	4.8 ± 1.0	4.4 ± 0.8	4.7 ± 0.8	4.9 ± 1.0	5.2 ± 1.2	<0.001
ALT, u/L	25.0 (17.0-40.0)	24.0 (16.0-39.0)	26.0 (17.0-39.0)	25.0 (17.8-40.2)	25.0 (16.0-40.0)	0.320
Glucose, mg/dl	505.0 (382.0-659.0)	418.5 (319.0-537.2)	497.0 (397.0-615.0)	563.5 (427.2-711.8)	576.0 (430.2-781.2)	<0.001
WBC, k/ul	14.8 ± 8.0	12.7 ± 6.1	14.7 ± 7.9	15.7 ± 8.2	16.3 ± 9.2	<0.001
eGFR, mL/min per 1.73 m^2^	61.0 (35.0-96.0)	103.0 (78.0-126.0)	77.0 (57.0-105.0)	50.0 (36.0-70.0)	24.0 (15.0-37.0)	<0.001
Hemoglobin, g/dl	13.5 ± 2.6	14.7 ± 2.1	14.3 ± 2.2	13.4 ± 2.4	11.8 ± 2.7	<0.001
Albumin, g/dl	3.7 ± 0.8	4.1 ± 0.7	4.0 ± 0.7	3.6 ± 0.7	3.0 ± 0.7	<0.001
Bicarbonate, mEq/L	16.0 ± 6.4	14.9 ± 6.0	16.0 ± 6.3	16.8 ± 6.6	16.2 ± 6.5	<0.001
**IVU, n (%)**	294 (4.4)	12 (1.3)	12 (1.3)	46 (5.1)	109 (12.1)	<0.001
**Dialysis, n (%)**	89 (2.5)	1 (0.1)	4 (0.4)	20 (2.2)	64 (7.1)	<0.001
**GCS**	13.5 ± 3.5	14.3 ± 2.7	14.1 ± 3.0	13.5 ± 3.6	12.3 ± 4.3	<0.001
**Death, n (%)**	86 (2.4)	4 (0.4)	4 (0.4)	20 (2.2)	58 (6.4)	<0.001

BMI, body mass index; MAP, mean arterial blood pressure; AMI: acute myocardial infarction; CHF, congestive heart failure; CKD, chronic kidney disease; BUN, blood urea nitrogen; ALT, alanine aminotransferase; WBC, white blood cell count; eGFR, estimated glomerular filtration rate; IVU Invasive ventilation use; GCS, Glasgow coma scale.

Starting with demographic variables, there was a steady increase in age (p<0.001) and percentage of males (p<0.001) from the first to the fourth quartile. The ethnicity showed differences in distribution (p=0.002). BMI, heart rates, and MAP also progressively inclined across the quartiles, indicating a significant variation (p<0.001).

Medical history revealed higher trends of pre-existing conditions, such as infectious disease, neurological diseases, AMI, hypertension, CHF, and CKD from Q1 to Q4 (p<0.001, for each). The laboratory results further corroborated these trends, with an overall increase across the quartiles in values of BUN, potassium, glucose, WBC, and significant reduction in eGFR (p<0.001 for each). Sodium, hemoglobin, and albumin levels also displayed dipping trends from Q1 to Q4 with statistical significance (p<0.001). Instances of IVU and dialysis seemed to escalate from Q1 to Q4 with a significant p-value (<0.001). A similar progression was noted for mortality rates across the quartiles (p<0.001).

### 3.2 Association between LnBAR and in-hospital death

**Figure 2:**
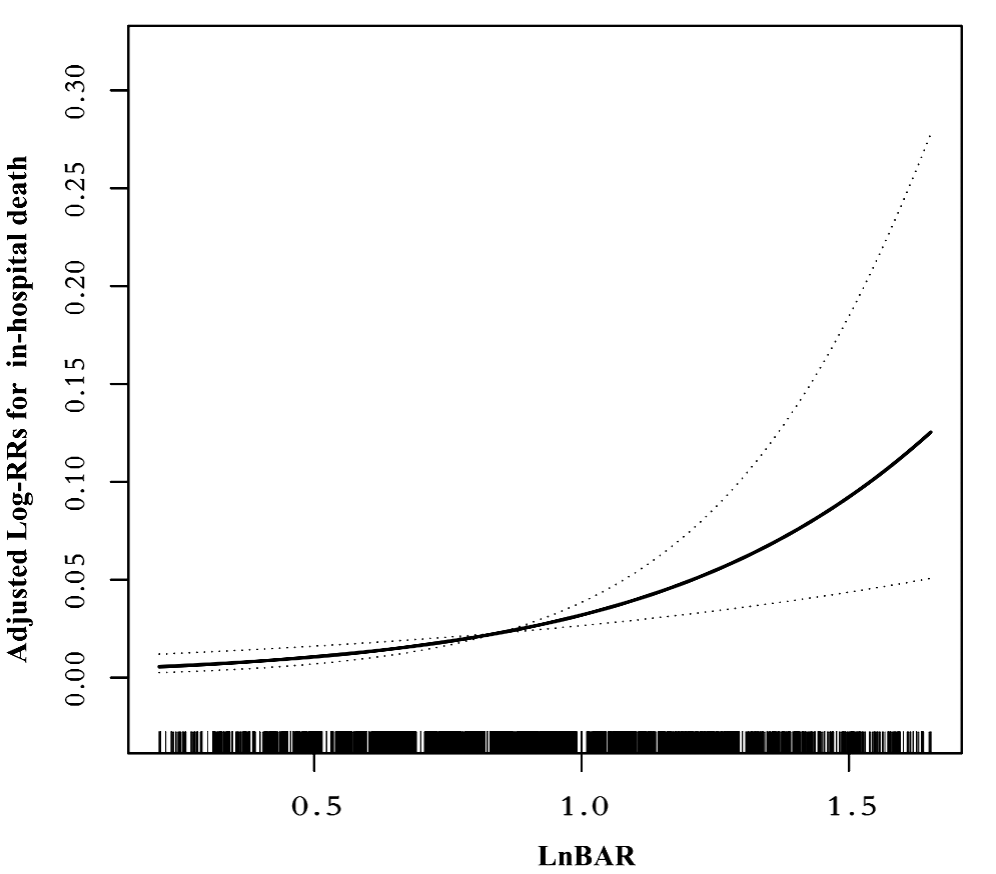
Nonlinear dose-response relationship between LnBAR and in-Hospital mortality. The adjusted variables were consistent with Model 2 in the multivariate regression analysis. The solid line and dashed line represent the estimated values and their corresponding 95% confidence intervals.

Following the Akaike information criterion, a total of 3 knots were used in modeling. Knot positions were selected at the 10th, 50th, and 90th percentiles of baseline LnBAR levels, among which the 50th percentile was selected as the reference value. In the unstratified cohort, a consistently significant nonlinear association (P for nonlinearity <0.012) between baseline LnBAR and in-hospital death was observed after adjusting for multiple confounders (**Figure 2**).

In the multivariable Cox regression analysis (**Table 2**), the association between LnBAR and in-hospital mortality was assessed across three different models. The LnBAR, as a continuous variable, showed a significant association with in-hospital mortality in the unadjusted model, Model 1, and Model 2, with hazard ratios (HRs) of 1.89 (95% confidence interval [CI], 1.54–2.34; P<0.001), 1.73 (95% CI, 1.38–2.16; P<0.001), and 1.82 (95% CI, 1.42–2.34; P<0.001), respectively.

**Table 2:** Multivariable Cox regression analysis to assess the association between LnBAR and in-hospital mortality in different models.

Variable	Unadjusted		Model 1		Model 2	
	HR 95% CI	*P*-value	HR 95% CI	*P*-value	HR 95% CI	*P*-value
LnBAR ^*^	1.89 (1.54, 2.34)	<0.001	1.73 (1.38, 3.16)	<0.001	1.82 (1.42, 2.34)	<0.001
Quartiles						
Q1 (BAR<4.07)	Reference		Reference		Reference	
Q2 (4.07≤BAR<6.28)	2.73 (0.87, 8.56)	0.086	2.4 (0.76, 7.57)	0.139	2.34 (0.74, 7.39)	0.151
Q3 (6.28≤BAR<11.58)	3.75 (1.3, 10.86)	0.015	2.57 (0.86, 7.61)	0.093	2.52 (0.85, 7.47)	0.1
Q4 (BAR≥11.58)	6.83 (2.47, 18.92)	<0.001	4.36 (1.52, 12.51)	0.007	4.29 (1.49, 12.34)	0.008
***P* for trend**		<0.001		0.001		0.001
Q1-Q2 (BAR<6.28)	Reference		Reference		Reference	
Q3-Q4 (BAR≥6.28)	2.95 (1.69, 5.15)	<0.001	2 (1.11, 3.62)	0.024	1.99 (1.1, 3.6)	0.025

BAR, Blood urea nitrogen to serum albumin ratio. *LnBAR was entered as a continuous variable. Crude model was adjusted for none. Model 1 = Adjusted for age, gender, ethnicity. Model 2 = Adjusted for Model 1 + (BMI, heart rate, and mean artery pressure).

When BAR was categorized into quartiles, the highest quartile (Q4, BAR > 11.58) had a significantly higher risk of in-hospital mortality compared with the reference first quartile (Q1, BAR < 4.07) in all three models: unadjusted (HR, 6.83; 95% CI, 2.47–18.67; P<0.001), Model 1 (HR, 4.36; 95% CI, 1.52–12.51; P=0.007), and Model 2 (HR, 4.29; 95% CI, 1.49–12.34; P=0.008). The third quartile (Q3, 6.28≤BAR<11.58) also demonstrated a significant association with in-hospital mortality in the unadjusted model (HR, 3.75; 95% CI, 1.3–10.86; P=0.015) but did not reach statistical significance in Model 1 and Model 2. Quartile 2 (Q2, 4.07≤BAR<6.28) did not show a significant increase in risk across all models.

Furthermore, a significant trend across increasing BAR quartiles was observed for in-hospital mortality (P for trend <0.001 in unadjusted model; P for trend = 0.001 in Model 1 and Model 2). When combining quartiles to compare groups below and above a BAR of 6.28, a significant association with in-hospital mortality was found for Q3-Q4 (BAR ≥ 6.28) versus Q1-Q2 (BAR < 6.28) with HRs of 2.95 (95% CI, 1.69–5.15; P<0.001) in the unadjusted model, 2 (95% CI, 1.11–3.62; P = 0.024) in Model 1, and 1.99 (95% CI, 1.1–3.6; P = 0.025) in Model 2.

### 3.3 Subgroup analysis

**Figure 3:**
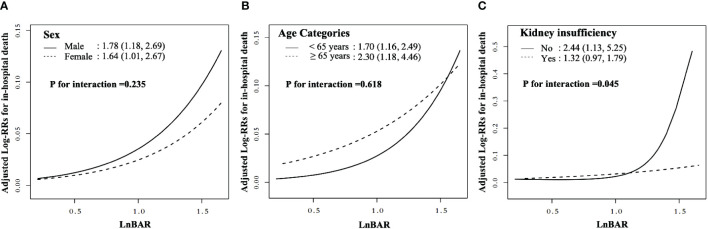
Nonlinear dose-response relationship between LnBAR and in-Hospital mortality in subgroup by sex, age, and kidney insufficiency. The adjusted variables were consistent with Model 2 in the multivariate regression analysis. The solid line and dashed line represent the estimated values and their corresponding 95% confidence intervals. **(A)** Subgroup by sex; **(B)** Subgroup by age; **(C)** Subgroup by kidney insufficiency.

In the assessment of potential effect modifiers for the relationship between LnBAR and the risk of in-hospital mortality among patients with DKA, sex and age did not significantly modify the association (P for interaction = 0.494 and 0.774, respectively) (**Figure 3**). However, the association was significantly modified by the presence of kidney insufficiency (P for interaction = 0.023). The HR for in-hospital death among patients without kidney insufficiency, for a per standard deviation increase in LnBAR, was 2.88 (95% CI, 1.71–4.85), which was notably higher than that among patients with kidney insufficiency at 1.35 (95% CI, 0.99–1.85). This indicates a stronger association of LnBAR with in-hospital mortality in patients without kidney insufficiency. The risk curves indicate that as LnBAR increases, the adjusted log RRs also increases, and this effect is more pronounced in patients without kidney insufficiency.

### 3.4 TDROC curve analysis for in-hospital mortality

**Figure 4:**
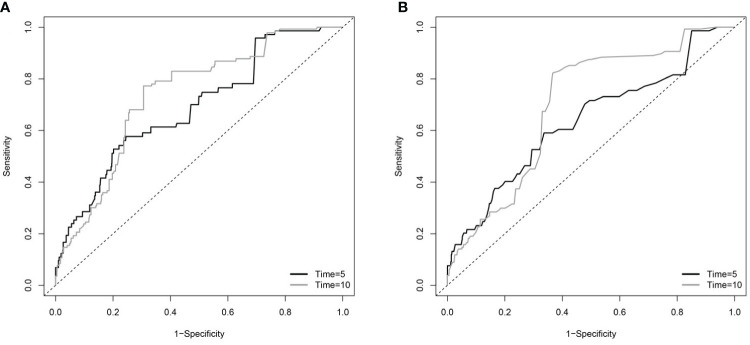
The time-dependent receiver operating characteristic (TDROC) curves of the predictive value of BAR and BUN for in-hospital mortality in patients with diabetes **(A)** TDROC for BAR. **(B)** TDROC for BUN. BAR, blood urea nitrogen to serum albumin ratio; BUN, blood urea nitrogen.

TDROC curves were used to compare the predictive value of BAR and BUN (**Figure 4** and **Table 3**). For BAR, for the 5-day mortality, the AUC was 0.6198, and the Max Youden was 0.2068. For 10-day mortality, the AUC was 0.6242, and the Max Youden was 0.2175. For BUN, for the 5-day mortality, the AUC was 0.6123, and the Max Youden was 0.1664. For 10-day mortality, the AUC was 0.6145, and the Max Youden was 0.175. Therefore, the predictive value of BAR appeared to perform better than BUN in terms of overall accuracy and balance between sensitivity and specificity for both 5-day mortality and 10-day mortality.

**Table 3:** The time-dependent receiver operating curves (TDROC) for the predictive value of BAR and BUN for in-hospital mortality in patients with diabetes.

	Time (days)	AUC	Max Youden
**BAR**	5	0.69	0.33
**BAR**	10	0.74	0.47
**BUN**	5	0.63	0.25
**BUN**	10	0.7	0.46

AUC, the area under the curve; BAR, blood urea nitrogen to serum albumin ratio; BUN, blood urea nitrogen.

### 3.5 Sensitivity analysis

The extent of missing data varied from 1.1% to 24.1% across the distinct variables. The distributions of any variable exhibiting missing data were the same between the imputation datasets and the observed complete case data (**Supplementary Table 1**). Multiple regression analyses using only subjects with complete data gave similar results to those undertaken on the multiple imputed datasets (**Supplementary Table 2**). Similar results were observed when further adjusting for serum electrolyte levels (including blood sodium, blood potassium, blood calcium, and blood chloride), indicators of liver and kidney function (eGFR, ALT, and AST), diabetes type and blood glucose level, blood gas parameters (blood bicarbonate, anion gap, PaO2 and chloride), indicators of liver and kidney function (eGFR, ALT, and AST), diabetes type and blood glucose level, blood gas parameters (blood bicarbonate, anion gap, PaO2, PaCO2), WBC count and hemoglobin level, sepsis and infectious disease, comorbidities (neurological disease, acute myocardial infarction, hypertension, heart failure, chronic renal failure), and GCS. (**Supplementary Table 3**). The results from data set without participants with eGFR < 30 ml/min/1.73m^2^ were consistent with the results from complete data set (**Supplementary Table 4**). The TDROC indicated that the predictive value of BAR appears to perform better than markers of acidosis including anion gap, bicarbonate, and blood glucose level in terms of overall accuracy and balance between sensitivity and specificity for both 5-day mortality and 10-day mortality (**Supplementary Table 5** and **Supplementary Figure 1**). The STROBE checklist of items in was shown in **Supplementary Table 6**.

## 4 Discussion

This study demonstrated that BAR was an independent risk factor for in-hospital death in ICU-admitted DKA patients. After adjusting for multiple confounders, LnBAR was nonlinearly associated with in-hospital death, with the risk of in-hospital death increasing with increasing LnBAR. The findings remained consistent in the unstratified cohort and the male cohort, and age-specific cohorts. However, in the groups based on kidney efficiency, we found that the relationship between LnBAR and in-hospital death was more pronounced in the group without kidney insufficiency. The results of the sensitivity analysis were consistent with the main analysis.

A previous study by Hang et al. demonstrated that elevated levels of BAR were associated with a higher risk of in-hospital mortality in critical DKA patients (25). Our study aimed to further investigate this relationship using a larger sample size and a multi-center approach. Based on sufficient participants, we grouped the data by quartiles, applied a trend test on the multivariate regression models, and visualized a fitted smooth curve. As a result, we further identified a nonlinear, monotonically increasing relationship between elevated BAR and in-hospital mortality of critical DKA patients. In addition, we conducted a subgroup analysis, which revealed a more pronounced relationship in the subgroup without kidney insufficiency. Therefore, our study bolsters previous research and further confirms the correlation between BAR and in-hospital mortality in critical DKA patients, enhancing its applicability in clinical settings.

The correlation between BAR and in-hospital mortality in patients with DKA may be associated with the pathophysiology intrinsic to DKA itself. DKA is often accompanied by volume depletion, which can lead to acute kidney injury (26, 27). BUN, as the end product of urea, is excreted by the kidneys and reflects the patient’s dehydration status (28, 29). It is also an indicator of renal failure (30, 31), surpassing other markers such as serum creatinine (32, 33).

On the other hand, there is a negative correlation between the patient’s serum albumin concentration and the risk of ketosis (27). Serum albumin is a reliable predictor of DKA occurrence in diabetic patients and an indicator of poor prognosis (34–36).Serum albumin levels may indirectly reflect the insulin reserves of diabetic patients, as they are inversely related to HbA1c (37). Serum albumin has a half-life of approximately 21 days, making it more sensitive to recent changes in mean blood glucose levels than HbA1C (38). This makes it a more suitable indicator of short-term variations in a patient’s blood glucose levels.

The study’s strengths include its large size, cohort design, multi-center data source, and consistent results in subgroups. However, our study has several limitations that should be acknowledged. Firstly, while the eICU database is a valuable resource, the quality of data may be variable due to potential inconsistencies and errors arising from the manual data entry across various ICU units from different hospitals. As such, we cannot completely rule out the possibility of inaccuracies in our data. Secondly, the generalizability of our findings may be limited as the eICU database primarily consists of data from the United States. Hence, our results may not be directly applicable to healthcare settings in other countries or to populations with diverse ethnic backgrounds. Thirdly, with the increased use of sodium-glucose cotransporter protein 2 inhibitor drugs, euglycemic diabetic ketoacidosis (EDKA) is becoming more common. We also observed a lower BAR in EDKA participants in analyses. However, since eICU data was collected between 2014 and 2015, when sodium-glucose cotransporter protein 2 inhibitor drugs were just becoming widely available, the number of EDKA cases was small in our cohort (181 participants, 3 in-hospital deaths). Further studies are necessary to validate the predictive value of BAR in the EDKA population. Fourthly, the complexity of the data precluded further analysis of trends in BAR during hospitalization and the impact of treatment regimens on admission mortality in patients with DKA. Further studies are necessary in the future to validate the impact of these factors on the predictive value of BAR, thereby further guiding the clinical application of BAR. Another significant limitation is the retrospective design of our study. While we have tried to control for confounding variables through statistical models, there remains the potential for selection bias and residual confounding. Additionally, this design prevents us from establishing a definitive cause-effect relationship. Furthermore, our study may have been affected by missing or incomplete data in the eICU database. Although we have attempted to address this issue by employing multiple imputations, this may still bias our results. Factors such as pH, plasma osmolality, and urine osmolality, which significantly impact the prognosis of DKA patients, were not included in our study due to numerous missing values in the eICU database. Furthermore, the categorization of ICU patients according to severity plays a crucial role in prognosis estimation. However, upon incorporating the APACHE IV scoring into our model, we observed multicollinearity. We conjecture that this occurrence is due to the inclusion of BUN and albumin within the APACHE IV scoring criteria. Additionally, the data of the SOFA score was not available within the eICU database. Finally, the database contains limited information on long-term follow-up, thereby limiting our understanding of the survival rates or long-term outcomes for the patients beyond their ICU stays. Accordingly, we recommend prospective studies and further research to validate and augment our findings, as well as mitigate these limitations.

## 5 Conclusion

In summary, the study indicated that baseline LnBAR is nonlinearly associated with in-hospital mortality among ICU-admitted DKA patients. After multiple adjustment, one unit increase in LnBAR was positively associated with the risk of in-hospital death. Furthermore, when BAR was categorized in quartiles, the higher risks of in-hospital death were found in participants in quartile 3 to 4 (BAR≥6.28), compared with those in quartile 1 to 2 (BAR<6.28). In the subgroup analysis, the LnBAR-hospital death association was significantly stronger in participants without kidney insufficiency. These highlighted associations provide a new insight into the prognosis of DKA patients and emphasize the potential importance of BAR as a predictive biomarker for in-hospital mortality among ICU-admitted DKA patients. Further studies are needed to validate these findings and to explore the precise mechanisms involved, which could pave the way for more targeted risk-reduction strategies in the future.

## Data availability statement

Publicly available datasets were analyzed in this study. This data can be found here: https://eicu-crd.mit.edu (elCU Collaborative Research Database).

## Author contributions

HC: Conceptualization, Funding acquisition, Investigation, Supervision, Writing – review & editing, Writing – original draft. YW: Funding acquisition, Investigation, Writing – original draft, Writing – review & editing. RJ: Methodology, Software, Supervision, Validation, Writing – review & editing. ML: Data curation, Formal analysis, Project administration, Resources, Software, Visualization, Writing – review & editing.
