# Supplementary material for: Association between blood urea nitrogen to serum albumin ratio and in-hospital mortality in critical patients with diabetic ketoacidosis: a retrospective analysis of the eICU database
Source: Front Endocrinol (Lausanne). 2024 Jun 27;15:1411891. doi: 10.3389/fendo.2024.1411891 (PMC11236567; doi:10.3389/fendo.2024.1411891)
Supplement: Supplementary file 1 [file DataSheet_1.docx]

Supplementary Material

**
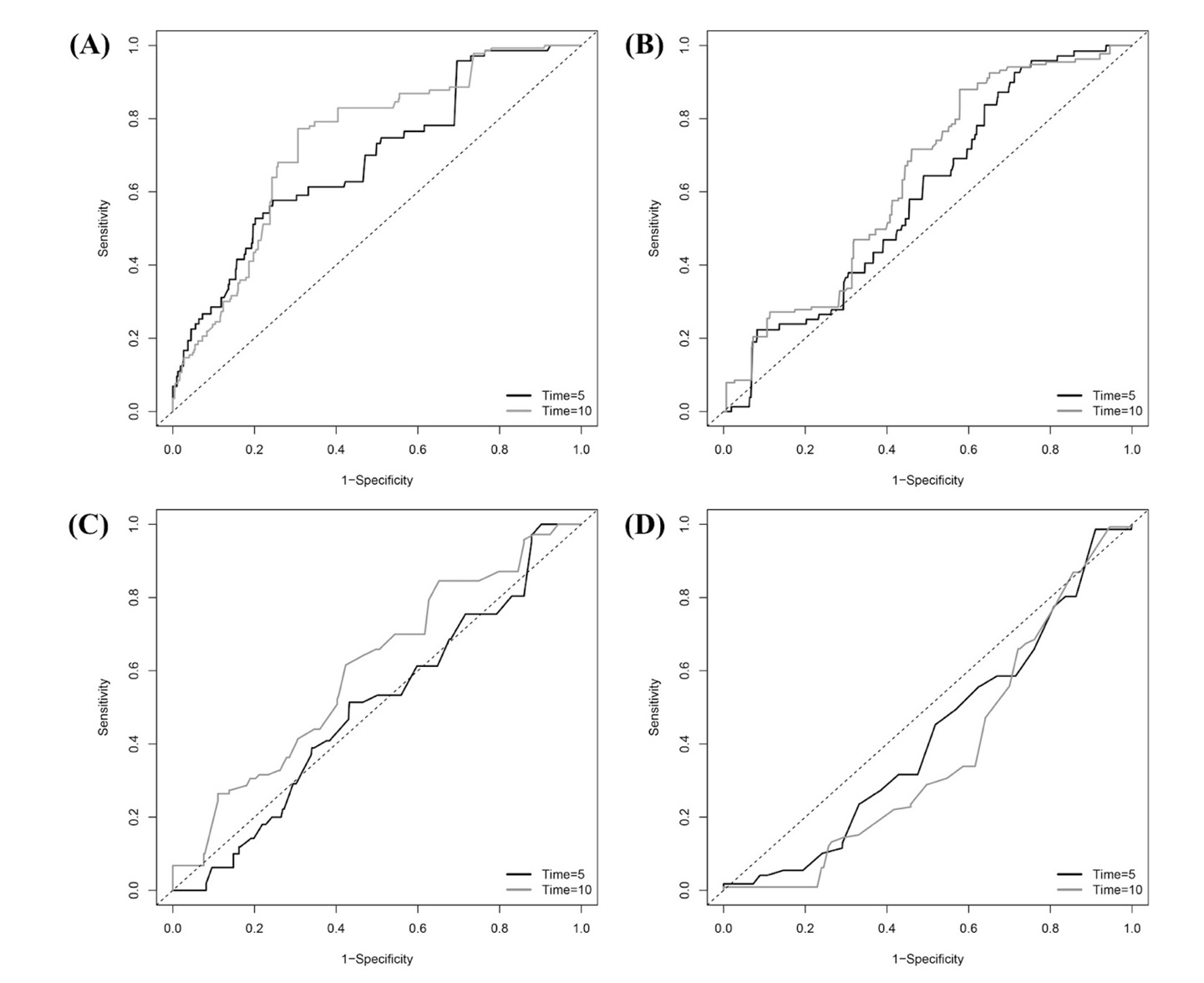
**

**Supplementary Figure 1.** The time-dependent receiver operating characteristic (TDROC) curves of the predictive value of BAR and markers of acidosis for in-hospital mortality in patients with diabetes (A) TDROC for BAR. (B) TDROC for blood glucose. (C) TDROC for anion gap; (D) TDROC for bicarbonate. BAR: blood urea nitrogen to serum albumin ratio.

**Supplementary Table 1.** Distributions of variables with missing data comparing observed complete case data to results from pooling the datasets with imputed variables from multiple imputation

| Variables | Level | Number (%) with missing data | Complete case | Multiple imputation |
| --- | --- | --- | --- | --- |
| Anionic gap | Mean ± standard deviation | 891 (24.1) | 23.9 ± 8.3 | 23.8 ± 7.4 |
| Net fluid balance in the first 24 hours | Median (interquartile range) | 889 (24.1) | 500.0 (240.0-800.0) | 544.9 (300.0-800.0) |
| BMI kg/m2 | Mean ± standard deviation | 40 (1.1) | 26.1 ± 7.4 | 26.1 ± 7.4 |
| ALT, u/L | Median (interquartile range) | 149 (4.0) | 25.0 (17.0-40.0) | 26.0 (17.0-40.0) |
| MAP, mmHg | Mean ± standard deviation | 104 (2.8) | 82.0 ± 36.2 | 82.0 ± 36.2 |
| Heart rate, bpm | Mean ± standard deviation | 104 (2.8) | 114.2 ± 23.9 | 114.2 ± 23.8 |
| GCS | Mean ± standard deviation | 0(0) | 13.5 ± 3.5 | 13.5 ± 3.5 |
| Bicarbonate, mEq/L | Mean ± standard deviation | 315(8.5) | 16.0 ± 6.4 | 15.9 ± 6.5 |

BMI, body mass index; ALT, alanine aminotransferase; MAP, mean arterial blood pressure; GCS, Glasgow coma scale.

**Supplementary Table 2.** Complete case-adjusted and multiple imputation-adjusted associations of LnBAR with in-hospital death.

| LnBAR |  | Complete case* | |  | Multiple imputation ^#^ | |
| --- | --- | --- | --- | --- | --- | --- |
|  |  | HR 95% CI | P-value |  | HR 95% CI | P-value |
| Per 1-SD increase* |  | 1.79 (1.32, 2.43) | <0.001 |  | 1.69 (1.22, 2.34) | <0.001 |
| Categories |  |  |  |  |  |  |
| Q1-Q2 (LnBAR < 0.8) |  | Reference |  |  | Reference |  |
| Q3-Q4 (LnBAR ≥ 0.8) |  | 2.46 (1.04, 5.79) | 0.039 |  | 3.79 (1.02, 14.09) | 0.047 |

BAR, Blood urea nitrogen to serum albumin ratio. LnBAR was entered as a continuous variable per 1-Standard deviation (SD). *Adjusted for age, gender, ethnicity, BMI, blood sodium blood potassium, ALT, eGFR, WBC, blood glucose, heart rate, MAP, infectious disease, neurological diseases, AMI, hypertension, CHF, CKD, GCS, bicarbonate, diabetes type, dialysis, and IVU. #Multiple imputation group further adjustment of the anion gap and the first 24-hour net fluid balance after multiple interpolation

**Supplementary Table 3.** Multivariable Cox regression analysis to assess the association between LnBAR and in-hospital mortality further adjusted for several covariates.

|  | **BAR quartiles** | | | | | **LnBAR** |
| --- | --- | --- | --- | --- | --- | --- |
| **Model** | **Q1 (BAR<4.07)** | **Q2 (4.07≤BAR<6.28)** | **Q3 (6.28≤BAR<11.58)** | **Q4 (BAR≥11.58)** |  | **Per 1 unit increment** |
| **Model 1** | Reference | 2.34 (0.74, 7.39) | 2.52 (0.85, 7.47) | 4.29 (1.49, 12.34) |  | 1.82 (1.42, 2.34) |
| **Model 2** | Reference | 2.3 (0.73, 7.28) | 2.44 (0.82, 7.31) | 4.18 (1.43, 12.21) |  | 1.85 (1.41, 2.41) |
| **Model 3** | Reference | 2.34 (0.73, 7.46) | 2.24 (0.72, 6.94) | 3.81 (1.21, 12) |  | 2.05 (1.48, 2.84) |
| **Model 4** | Reference | 2.48 (0.78, 7.84) | 2.5 (0.84, 7.47) | 4.18 (1.44, 12.12) |  | 1.8 (1.4, 2.31) |
| **Model 5** | Reference | 2.39 (0.75, 7.6) | 2.27 (0.75, 6.89) | 3.46 (1.2, 10.01) |  | 1.66 (1.3, 2.12) |
| **Model 6** | Reference | 2.16 (0.68, 6.84) | 2.23 (0.75, 6.66) | 3.24 (1.1, 9.57) |  | 1.64 (1.25, 2.16) |
| **Model 7** | Reference | 2.24 (0.71, 7.09) | 2.13 (0.71, 6.36) | 3.66 (1.26, 10.62) |  | 1.7 (1.32, 2.18) |
| **Model 8** | Reference | 2.53 (0.8, 8) | 2.17 (0.73, 6.51) | 3.89 (1.35, 11.22) |  | 1.71 (1.33, 2.21) |
| **Model 9** | Reference | 2.3 (0.73, 7.28) | 2.09 (0.7, 6.24) | 3.02 (1.03, 8.81) |  | 1.69 (1.3, 2.21) |
| **Model 10** | Reference | 2.34 (0.74, 7.4) | 2.26 (0.76, 6.69) | 3.41 (1.18, 9.83) |  | 1.66 (1.28, 2.15) |

Model 1 was adjusted for age, gender, ethnicity, body mass index, heart rate, and mean artery pressure.

Model 2: Model 1 + (blood sodium, blood potassium, blood calcium, and blood chloride)

Model 3: Model 1 + (ALT, AST, eGFR)

Model 4: Model 1 + (diabetes type, blood glucose level)

Model 5: Model 1 + (bicarbonate, anion gap, PaO_2_, PaCO_2_)

Model 6: Model 1 + (hemoglobin, white blood cell)

Model 7: Model 1 + (sepsis, infectious disease)

Model 8: Model 1 + (neurological diseases, acute myocardial infarction, hypertension, heart failure, chronic kidney dysfunction)

Model 9: Model 1 + (dialysis, invasive ventilation use)

Model 10: Model 1 + (Glasgow coma scale)

**Supplementary Table 4.** Multivariable Cox regression analysis to assess the association between LnBAR and in-hospital mortality in different models among diabetic patients without patients with eGFR <30 ml/min/1.73m2.

| Variable | Unadjusted | |  | Model 1 | |  | Model 2 | |
| --- | --- | --- | --- | --- | --- | --- | --- | --- |
|  | HR 95% CI | P-value |  | HR 95% CI | P-value |  | HR 95% CI | P-value |
| LnBAR | 2.89 (1.87~4.44) | <0.001 |  | 2.63 (1.66~4.17) | <0.001 |  | 2.77 (1.71~4.49) | <0.001 |
| Quartiles |  |  |  |  |  |  |  |  |
| Q1 (BAR<4.07) | Reference |  |  | Reference |  |  | Reference |  |
| Q2 (4.07≤BAR<6.28) | 0.74 (0.17~3.32) | 0.698 |  | 0.76 (0.17~3.44) | 0.728 |  | 0.74 (0.16~3.35) | 0.7 |
| Q3 (6.28≤BAR<11.58) | 3.25 (1.09~9.7) | 0.04 |  | 2.93 (0.94~9.09) | 0.07 |  | 2.83 (0.9~8.84) | 0.082 |
| Q4 (BAR≥11.58) | 6.35 (2.16~18.6) | 0.002 |  | 5.3 (1.72~16.38) | 0.006 |  | 4.81 (1.54~15.02) | 0.01 |
| P for trend |  | <0.001 |  |  | 0.001 |  |  | 0.001 |
| Q1-Q2 (BAR<6.28) | Reference |  |  | Reference |  |  | Reference |  |
| Q3-Q4 (BAR≥6.28) | 5.14 (2.28~11.58) | <0.001 |  | 4.36 (1.86~10.21) | 0.001 |  | 4.17 (1.78~9.78) | 0.002 |

BAR, Blood urea nitrogen to serum albumin ratio. *LnBAR was entered as a continuous variable per 1-Standard deviation (SD). Crude model was adjusted for none. Model 1 = Adjusted for age, gender, ethnicity. Model 2 = Adjusted for Model 1 + (BMI, heart rate, and mean artery pressure).

**Supplementary Table 5.** The time-dependent receiver operating curves (TDROC) for the predictive value of BAR and BUN for in-hospital mortality in patients with diabetes.

|  | **Time (days)** | **AUC** | **Max Youden** |
| --- | --- | --- | --- |
| **BAR** | 5 | 0.69 | 0.33 |
| **BAR** | 10 | 0.74 | 0.47 |
| **Blood glucose** | 5 | 0.59 | 0.21 |
| **Blood glucose** | 10 | 0.64 | 0.30 |
| **Anion gap** | 5 | 0.5 | 0.1 |
| **Anion gap** | 10 | 0.6 | 0.19 |
| **Bicarbonate** | 5 | 0.42 | 0.08 |
| **bicarbonate** | 10 | 0.38 | 0.05 |

AUC, the area under the curve; BAR: blood urea nitrogen to serum albumin ratio.

**Supplementary Table 6.** STROBE checklist of items.

| **Items** | **Item No** |  |
| --- | --- | --- |
| Title and abstract | 1 | Association between blood urea nitrogen to serum albumin ratio and in-hospital mortality in critical patients with diabetic ketoacidosis: A retrospective analysis of the eICU database |
|  |  | Cohort study |
|  |  | Abstract  Background: This study aimed to investigate the association between blood urea nitrogen to serum albumin ratio (BAR) and the risk of in-hospital mortality in patients with diabetic ketoacidosis.  Methods: A total of 3,962 diabetic ketoacidosis patients from the eICU Collaborative Research Database were included in this analysis. The primary outcome was in-hospital death.  Results: Over a median length of hospital stay of 3.1 days, 86 in-hospital deaths were identified. One unit increase in LnBAR was positively associated with the risk of in-hospital death (hazard ratio [HR], 1.82 [95% CI, 1.42-2.34]). Furthermore, a nonlinear, consistently increasing correlation between elevated BAR and in-hospital mortality was observed (P for trend =0.005 after multiple-adjusted). When BAR was categorized into quartiles, the higher risk of in-hospital death (multiple-adjusted HR, 1.99 [95% CI, (1.1-3.6)]) was found in participants in quartiles 3 to 4 (BAR≥6.28) compared with those in quartiles 1 to 2 (BAR<6.28). In the subgroup analysis, the LnBAR-hospital death association was significantly stronger in participants without kidney insufficiency (yes versus no, P-interaction=0.023).  Conclusions: There was a significant and positive association between BAR and the risk of in-hospital death in patients with diabetic ketoacidosis. Notably, the strength of this association was intensified among those without kidney insufficiency. |
| Introduction | | |
| Background/rationale | 2 | Blood urea nitrogen to serum albumin ratio (BAR), as an innovative risk factor, is readily identifiable and easily accessible. It has been established as a prognostic marker for severe chronic obstructive pulmonary disease, sepsis, and cerebral haemorrhage. Due to hyperglycaemia, DKA patients frequently exhibit osmotic diuresis, volume depletion, electrolyte imbalances, and a catabolic state. These conditions frequently result in complications such as dehydration, impaired renal function, and malnutrition. The BUN level and serum albumin level are closely related to these conditions and complications. Consequently, BAR, a novel indicator that combines both indicators, may be a valuable and easily accessible prognostic indicator for DKA. However, relevant studies are lacking. |
| Objectives | 3 | The aim of this study was to investigate the predictive value of BAR in relation to in-hospital mortality among patients with DKA. |
| Methods | | |
| Study design | 4 | Cohort study. |
| Setting | 5 | Research data were extracted from the eICU Collaborative Research Database, which included medical information on 200,859 ICU admissions for 139,367 unique patients who were admitted to 335 ICUs across 208 hospitals in the USA between 2014 and 2015. |
| Participants | 6 | A total of 4,441 DKA patients were initially identified by looking for International Classification of Diseases version 9 (ICD-9) code 250.1 (Diabetes with ketoacidosis) and the text “DKA/diabetic ketoacidosis” in the diagnosis datafile. The exclusion criteria included: (1) discharged within 4 hours after ICU admission; (2) no laboratory record of BUN or albumin results during the first 24 hours or after ICU admission. For patients with multiple ICU admissions, only the first admission record was kept. A total of 3,962 DKA patients entered the study cohort. |
| Variables | 7 | Exposure: The natural logarithm of BAR (LnBAR) of patients measured at the time closest to the ICU admission time and within 24 h of ICU admission was employed.  Outcome: In-hospital death.  Covariates: Body mass index. Ethnicity. Comorbidities including diabetes type, hypertension, acute myocardial infarction, heart failure, infectious disease, chronic kidney disease, neurological diseases, dialysis requirement, and history of invasive ventilation use (IVU). The vital signs including heart rate and mean arterial pressure were included. Serum electrolyte levels including blood sodium, blood potassium, blood calcium, and blood chloride were included. Indicators of liver and kidney function including eGFR, alanine transferase (ALT), and aspartate aminotransferase (AST) were included. The level of blood glucose was included. Blood gas parameters including bicarbonate, anion gap, PaO2, and PaCO2 were included. White blood cell counts and haemoglobin levels in the blood were included. Sepsis was included. The treatment histories of dialysis and invasive ventilation were included. Furthermore, Glasgow Coma Scale (GCS) was included to identify patients at high risk. |
| Data sources/ measurement | 8* | The variables were measured as described above. |
| Bias | 9 | To circumvent the effects of missing values, multiple imputation was employed. Multiple COX regression models were constructed to adjust for the potential effects of covariates. Restricted cubic spline plots were utilized to assess the existence of any nonlinear relationships. |
| Study size | 10 | Based on general recommendations for COX proportional risk modelling, each covariate adjusted corresponds to 10-15 events. |
| Quantitative variables | 11 | In analyses, the quantitative variables were treated as continuous variables. |
| Statistical methods | 12 | (a) Baseline characteristics of the study cohort were stratified by the quartile of their baseline LnBAR levels. Continuous data were presented as the mean (standard deviation [SD]) for normally distributed data and the median (interquartile range [IQR]) for skewed data. Categorical data were presented as the total number of an item with column percentage (n [%]) unless otherwise specified. Crude comparisons between strata were performed using Kruskal-Wallis rank test for continuous data and chi-square tests for categorical variables.  (b) The adjusted logarithmic relative risks (logRRs) of in-hospital death plotted against LnBAR were visualized, with a fitted smoothed curve to represent the trend. Non-linearity of these associations was tested with a likelihood ratio test. we used a recursive algorithm to calculate the inflection points.  (c) Univariate and multivariate Cox regression models were used to assess the hazard ratio (HR) of LnBAR with in-hospital mortality. |
|  |  | (a) We graphically represented the adjusted log RRs for in-hospital death across the continuous range of LnBAR using three separate plots stratified by sex, age categories (<65 years vs. ≥65 years), and kidney insufficiency status (yes vs. no). |
|  |  | (a) To maintain statistical power and diminish bias that might occur after subjects with missing data were excluded from analyses, we used multivariate multiple imputation with chained equations to impute missing values. |
|  |  | (a) Sensitivity analysis was performed. (1) To authenticate the reliability of the data following multiple imputations, the percentage accounted for by the missing variables were computed. Subsequently, we orchestrated a comparison of the distributions of the missing variables prior to and following the implementation of multiple imputation. (2) Moreover, multivariate Cox regression was performed on complete case data as well as the dataset post multiple imputation separately. (3) To investigate a potential role of serum electrolyte levels, liver and kidney indices, diabetes type and blood glucose level, blood gas parameters, WBC count and hemoglobin level, sepsis and infectious diseases, comorbidities, and GCS with any of the observed associations, we further adjusted for serum electrolyte levels (including blood sodium, blood potassium, blood calcium, and blood chloride), indicators of liver and kidney function (eGFR, ALT, and AST), diabetes type and blood glucose level, blood gas parameters (blood bicarbonate, anion gap, PaO2 and chloride), indicators of liver and kidney function (eGFR, ALT, and AST), diabetes type and blood glucose level, blood gas parameters (blood bicarbonate, anion gap, PaO2, PaCO2), WBC count and hemoglobin level, sepsis and infectious disease, comorbidities (neurological disease, acute myocardial infarction, hypertension, heart failure, chronic renal failure), and GCS. (4) The main analyses were repeated on data set without participants with eGFR < 30 ml/min/1.73m2. (5) The areas under the TDROC curves were employed to assess the predictive value of BAR in comparison to that of other direct markers of acidosis, including serum anion gap, serum bicarbonate, and blood glucose. |

| Results | | |
| --- | --- | --- |
| Participants | 13* | 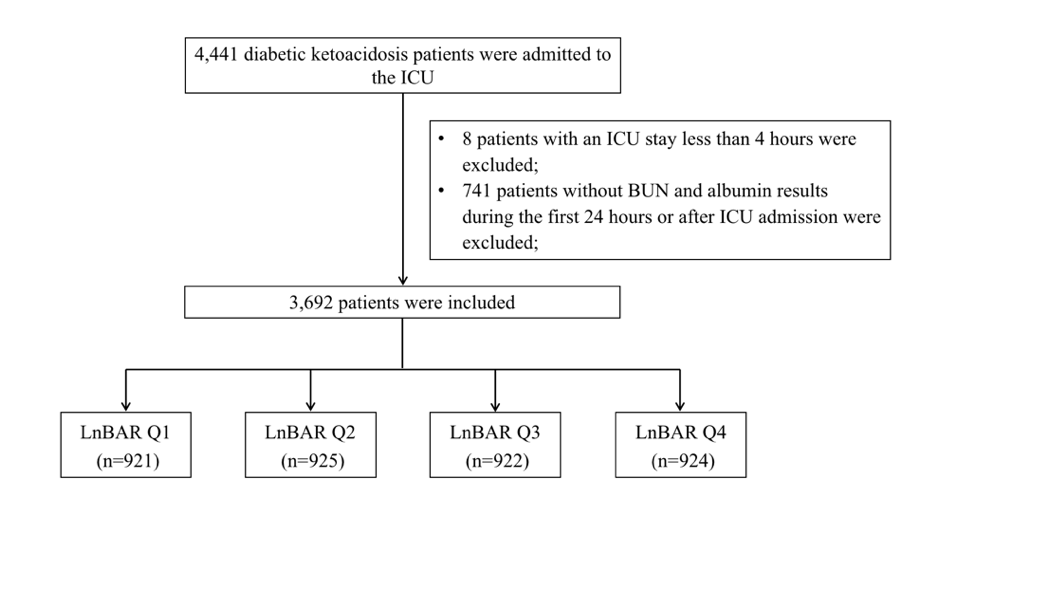 |
| Descriptive data | 14* | Significant differences in baseline characteristics including age, sex, ethnicity, BMI, heart rate, MAP, medical history, laboratory results, IVU, and dialysis were found among baseline BAR quartiles (Table 1).  Starting with demographic variables, there was a steady increase in age (p<0.001) and percentage of males (p<0.001) from the first to the fourth quartile. The ethnicity showed differences in distribution (p=0.002). BMI, heart rates, and MAP also progressively inclined across the quartiles, indicating a significant variation (p<0.001).  Medical history revealed higher trends of pre-existing conditions, such as infectious disease, neurological diseases, AMI, hypertension, CHF, and CKD from Q1 to Q4 (p<0.001, for each). The laboratory results further corroborated these trends, with an overall increase across the quartiles in values of BUN, potassium, glucose, WBC, and significant reduction in eGFR (p<0.001 for each). Sodium, hemoglobin, and albumin levels also displayed dipping trends from Q1 to Q4 with statistical significance (p<0.001). Instances of IVU and dialysis seemed to escalate from Q1 to Q4 with a significant p-value (<0.001). A similar progression was noted for mortality rates across the quartiles (p<0.001). |
| Outcome data | 15* | Over a median length of hospital stay of 3.1 days, 86 in-hospital deaths were identified. |
| Main results | 16 | In the multivariable Cox regression analysis, the association between LnBAR and in-hospital mortality was assessed across three different models. The LnBAR, as a continuous variable, showed a significant association with in-hospital mortality in the unadjusted model, Model 1, and Model 2, with hazard ratios (HRs) of 1.89 (95% confidence interval [CI], 1.54-2.34; P<0.001), 1.73 (95% CI, 1.38-2.16; P<0.001), and 1.82 (95% CI, 1.42-2.34; P<0.001), respectively.  When BAR was categorized into quartiles, the highest quartile (Q4, BAR > 11.58) had a significantly higher risk of in-hospital mortality compared with the reference first quartile (Q1, BAR < 4.07) in all three models: unadjusted (HR, 6.83; 95% CI, 2.47-18.67; P<0.001), Model 1 (HR, 4.36; 95% CI, 1.52-12.51; P=0.007), and Model 2 (HR, 4.29; 95% CI, 1.49-12.34; P=0.008). The third quartile (Q3, 6.28≤BAR<11.58) also demonstrated a significant association with in-hospital mortality in the unadjusted model (HR, 3.75; 95% CI, 1.3-10.86; P=0.015) but did not reach statistical significance in Model 1 and Model 2. Quartile 2 (Q2, 4.07≤BAR<6.28) did not show a significant increase in risk across all models.  Furthermore, a significant trend across increasing BAR quartiles was observed for in-hospital mortality (P for trend <0.001 in unadjusted model; P for trend = 0.001 in Model 1 and Model 2). When combining quartiles to compare groups below and above a BAR of 6.28, a significant association with in-hospital mortality was found for Q3-Q4 (BAR ≥ 6.28) versus Q1-Q2 (BAR < 6.28) with HRs of 2.95 (95% CI, 1.69-5.15; P<0.001) in the unadjusted model, 2 (95% CI, 1.11-3.62; P = 0.024) in Model 1, and 1.99 (95% CI, 1.1-3.6; P = 0.025) in Model 2. |
| Other analyses | 17 | Subgroups analyses:  In the assessment of potential effect modifiers for the relationship between LnBAR and the risk of in-hospital mortality among patients with DKA, sex and age did not significantly modify the association (P for interaction = 0.494 and 0.774, respectively) (Figure 3). However, the association was significantly modified by the presence of kidney insufficiency (P for interaction = 0.023). The HR for in-hospital death among patients without kidney insufficiency, for a per standard deviation increase in LnBAR, was 2.88 (95% CI, 1.71-4.85), which was notably higher than that among patients with kidney insufficiency at 1.35 (95% CI, 0.99-1.85). This indicates a stronger association of LnBAR with in-hospital mortality in patients without kidney insufficiency. The risk curves indicate that as LnBAR increases, the adjusted log RRs also increases, and this effect is more pronounced in patients without kidney insufficiency.  TDROC curve analysis for in-hospital mortality:  TDROC curves were used to compare the predictive value of BAR and BUN (Figure 5 and Table 7). For BAR, for the 5-day mortality, the AUC was 0.6198, and the Max Youden was 0.2068. For 10-day mortality, the AUC was 0.6242, and the Max Youden was 0.2175. For BUN, for the 5-day mortality, the AUC was 0.6123, and the Max Youden was 0.1664. For 10-day mortality, the AUC was 0.6145, and the Max Youden was 0.175. Therefore, the predictive value of BAR appeared to perform better than BUN in terms of overall accuracy and balance between sensitivity and speciﬁcity for both 5-day mortality and 10-day mortality.  Sensitivity analysis:  The extent of missing data varied from 1.1% to 24.1% across the distinct variables. The distributions of any variable exhibiting missing data were the same between the imputation datasets and the observed complete case data. Multiple regression analyses using only subjects with complete data gave similar results to those undertaken on the multiple imputed datasets. Similar results were observed when further adjusting for serum electrolyte levels (including blood sodium, blood potassium, blood calcium, and blood chloride), indicators of liver and kidney function (eGFR, ALT, and AST), diabetes type and blood glucose level, blood gas parameters (blood bicarbonate, anion gap, PaO2 and chloride), indicators of liver and kidney function (eGFR, ALT, and AST), diabetes type and blood glucose level, blood gas parameters (blood bicarbonate, anion gap, PaO2, PaCO2), WBC count and hemoglobin level, sepsis and infectious disease, comorbidities (neurological disease, acute myocardial infarction, hypertension, heart failure, chronic renal failure), and GCS. The results from data set without participants with eGFR < 30 ml/min/1.73m2 were consistent with the results from complete data set. The TDROC indicated that the predictive value of BAR appears to perform better than markers of acidosis including anion gap, bicarbonate, and blood glucose level in terms of overall accuracy and balance between sensitivity and specificity for both 5-day mortality and 10-day mortality. |
| Discussion | | |
| Key results | 18 | This study demonstrated that BAR was an independent risk factor for in-hospital death in ICU-admitted DKA patients. After adjusting for multiple confounders, LnBAR was nonlinearly associated with in-hospital death, with the risk of in-hospital death increasing with increasing LnBAR. The findings remained consistent in the unstratified cohort and the male cohort, and age-specific cohorts. However, in the groups based on kidney efficiency, we found that the relationship between LnBAR and in-hospital death was more pronounced in the group without kidney insufficiency. The results of the sensitivity analysis were consistent with the main analysis. |
| Limitations | 19 | Our study has several limitations that should be acknowledged. Firstly, while the eICU database is a valuable resource, the quality of data may be variable due to potential inconsistencies and errors arising from the manual data entry across various ICU units from different hospitals. As such, we cannot completely rule out the possibility of inaccuracies in our data. Secondly, the generalizability of our findings may be limited as the eICU database primarily consists of data from the United States. Hence, our results may not be directly applicable to healthcare settings in other countries or to populations with diverse ethnic backgrounds. Thirdly, with the increased use of sodium-glucose cotransporter protein 2 inhibitor drugs, euglycemic diabetic ketoacidosis (EDKA) is becoming more common. We also observed a lower BAR in EDKA participants in analyses. However, since eICU data was collected between 2014 and 2015, when sodium-glucose cotransporter protein 2 inhibitor drugs were just becoming widely available, the number of EDKA cases was small in our cohort (181 participants, 3 in-hospital deaths). Further studies are necessary to validate the predictive value of BAR in the EDKA population. Fourthly, the complexity of the data precluded further analysis of trends in BAR during hospitalization and the impact of treatment regimens on admission mortality in patients with DKA. Further studies are necessary in the future to validate the impact of these factors on the predictive value of BAR, thereby further guiding the clinical application of BAR. Another significant limitation is the retrospective design of our study. While we have tried to control for confounding variables through statistical models, there remains the potential for selection bias and residual confounding. Additionally, this design prevents us from establishing a definitive cause-effect relationship. Furthermore, our study may have been affected by missing or incomplete data in the eICU database. Although we have attempted to address this issue by employing multiple imputations, this may still bias our results. Factors such as pH, plasma osmolality, and urine osmolality, which significantly impact the prognosis of DKA patients, were not included in our study due to numerous missing values in the eICU database. Furthermore, the categorization of ICU patients according to severity plays a crucial role in prognosis estimation. However, upon incorporating the APACHE IV scoring into our model, we observed multicollinearity. We conjecture that this occurrence is due to the inclusion of BUN and albumin within the APACHE IV scoring criteria. Additionally, the data of the SOFA score was not available within the eICU database. Finally, the database contains limited information on long-term follow-up, thereby limiting our understanding of the survival rates or long-term outcomes for the patients beyond their ICU stays. Accordingly, we recommend prospective studies and further research to validate and augment our findings, as well as mitigate these limitations. |
| Interpretation | 20 | The study indicated that baseline LnBAR is nonlinearly associated with in-hospital mortality among ICU-admitted DKA patients. |
| Generalisability | 21 | These highlighted associations provide a new insight into the prognosis of DKA patients and emphasize the potential importance of BAR as a predictive biomarker for in-hospital mortality among ICU-admitted DKA patients. Further studies are needed to validate these findings and to explore the precise mechanisms involved, which could pave the way for more targeted risk-reduction strategies in the future. |
| Other information | | |
| Funding | 22 | The authors declare that the research was conducted in the absence of any commercial or financial relationships that could be construed as a potential conflict of interest. |

STROBE: STrengthening the Reporting of OBservational studies in Epidemiology.
